# Robot-Assisted Training of the Kinesthetic Sense: Enhancing Proprioception after Stroke

**DOI:** 10.3389/fnhum.2014.01037

**Published:** 2015-01-05

**Authors:** Dalia De Santis, Jacopo Zenzeri, Maura Casadio, Lorenzo Masia, Assunta Riva, Pietro Morasso, Valentina Squeri

**Affiliations:** ^1^Motor Learning and Robotic Rehabilitation Laboratory, Department of Robotics, Brain and Cognitive Sciences (RBCS), Istituto Italiano di Tecnologia, Genova, Italy; ^2^NeuroLab, Department of Informatics, Bioengineering, Robotics and Systems (DIBRIS), University of Genova, Genova, Italy; ^3^Assistive Robotics and Interactive Ergonomic Systems Laboratory, Division of Mechatronics and Design, Robotic Research Center, School of Mechanical and Aerospace Engineering (MAE), Nanyang Technological University (NTU), Singapore; ^4^SI4LIFE – Innovation Hub for Elderly and Disabled People, Genova, Italy

**Keywords:** haptic interaction, proprioception, force perception, robot assistance, kinesthetic acuity, robot therapy, pulsed assistance, stroke survivors

## Abstract

Proprioception has a crucial role in promoting or hindering motor learning. In particular, an intact position sense strongly correlates with the chances of recovery after stroke. A great majority of neurological patients present both motor dysfunctions and impairments in kinesthesia, but traditional robot and virtual reality training techniques focus either in recovering motor functions or in assessing proprioceptive deficits. An open challenge is to implement effective and reliable tests and training protocols for proprioception that go beyond the mere position sense evaluation and exploit the intrinsic bidirectionality of the kinesthetic sense, which refers to both sense of position and sense of movement. Modulated haptic interaction has a leading role in promoting sensorimotor integration, and it is a natural way to enhance volitional effort. Therefore, we designed a preliminary clinical study to test a new proprioception-based motor training technique for augmenting kinesthetic awareness via haptic feedback. The feedback was provided by a robotic manipulandum and the test involved seven chronic hemiparetic subjects over 3 weeks. The protocol included evaluation sessions that consisted of a psychometric estimate of the subject’s kinesthetic sensation, and training sessions, in which the subject executed planar reaching movements in the absence of vision and under a minimally assistive haptic guidance made by sequences of graded force pulses. The bidirectional haptic interaction between the subject and the robot was optimally adapted to each participant in order to achieve a uniform task difficulty over the workspace. All the subjects consistently improved in the perceptual scores as a consequence of training. Moreover, they could minimize the level of haptic guidance in time. Results suggest that the proposed method is effective in enhancing kinesthetic acuity, but the level of impairment may affect the ability of subjects to retain their improvement in time.

## Introduction

In recent years, it has become evident that proprioception has a crucial role in promoting or hindering motor learning (Ostry et al., 2010; Wong et al., 2012; Vahdat et al., 2014). In particular, it has been shown that an intact position sense following stroke strongly correlates with the likelihood of motor recovery of the hemiplegic arm (Kusoffsky et al., 1982; Smith et al., 1983; Rand et al., 1999; Schabrun and Hillier, 2009). This is further supported by the recent neurophysiological finding that sensory input is integral in the preservation of cortical representation in both motor and sensory areas (Schabrun and Hillier, 2009; Chieffo et al., 2013; Yarossi et al., 2014). Indeed, the absence or reduction of sensory input in stroke subjects is known to bring about learned non-use and impaired or lost ability to react to or process sensory stimuli in the space contralateral to the brain lesion, a condition referred to as unilateral spatial neglect (Taub and Berman, 1963; Kerkhoff and Rossetti, 2006). The functional impairment resulting from a decreased sensory awareness severely impacts on the quality of life of stroke survivors. The impaired spontaneous use of the affected limb, the inability to maintain a sustained grasp and manipulate objects without vision, and the reduced ability to reacquire skilled movements limit the independence in daily life activities (Carey et al., 1993; Tyson et al., 2008; Meyer et al., 2014). The integrity of the somatosensory function was found to have a prognostic impact on rehabilitation interventions and overall recovery not only after stroke but also for other categories of neurological impairments like traumatic brain injury, spinal cord injury, and multiple sclerosis (for a brief review, see Bowerman et al., 2012).

Post-stroke sensory dysfunction occurs on average in one over two stroke survivors (Sullivan and Hedman, 2008), 17–52% manifesting specific impairments in proprioception (Doyle et al., 2010; Dukelow et al., 2012), percentage that increased to 34–64% when assessed through the Nottingham Sensory Assessment (Connell et al., 2008). Despite a great majority of neurological patients presenting both motor dysfunctions and kinesthetic deficits, sensory retraining after stroke is often disregarded by current rehabilitation protocols. Passive sensory training techniques, such as cutaneous electrical stimulation, were found to have significant beneficial effects on hand function as assessed by clinical rating scales (Schabrun and Hillier, 2009). Nevertheless, it is known that proprioception and in particular kinesthetic acuity is probably of greater functional value when subjects are active rather than passive (Gandevia et al., 1992). Moreover, current theories of perceptual learning and recovery of function in people with brain damage recommend promoting active participation of the subjects rather than passive mobilization in conjunction with the use of meaningful and accurate feedback (Morasso, 2013). Surprisingly, also traditional robot and virtual reality training techniques focus either in recovering motor functions or in assessing proprioceptive deficits with no attempts to apply haptic feedback to specifically enhance proprioception, with the only exception of Squeri et al. (2011).

Undamaged or healthy brain neuronal connections and cortical maps are continuously remodeled by experience and by the performance of specific, intensive, and complex movements used to solve motor problems and attain goals (activity-dependent plasticity, Fisher and Sullivan, 2001). Since neuroplastic changes following stroke may rapidly bring to functional limitation due to learned non-use and compensatory behaviors related to overreliance on the unaffected limbs, it is fundamental to devise interventions that involve the affected extremities into (voluntary) activity. Stroke recovery appears to be dependent upon skill learning rather than simple adaptation mechanisms (Dipietro et al., 2012). This process can be mediated by the use of robots to provide a very specific and intensive interactive practice (Turner et al., 2013). Indeed, recent studies have shown that robot-mediated training is able to induce neuroplastic changes in the brain (Pellegrino et al., 2012; Kantak et al., 2013; Milot et al., 2014). A second great advantage of robots for rehabilitation is the possibility to increase the stimulation of afferent pathways by providing graded tactile and force feedback (FF). Since neuroplasticity is experience-driven, interventions to promote neurorecovery should attend to both motor skill learning and its sensory consequences. There is recent evidence that motor learning drives functional reorganization of the brain that also affects sensory areas (Ostry et al., 2010). In turn, perceptual learning leads to persistent changes in motor areas in the brain (Nasir et al., 2013). The mechanisms beyond restoration of the kinesthetic sense are still poorly understood, but previous research support the hypothesis that kinesthetic training may be beneficial both to motor and perceptual aspects of the movement after stroke.

The open challenge is to devise new methods for retraining or enhancing kinesthetic sensation that take advantage of brain plasticity to restore the connection between action and perception intrinsic in kinesthesia, since it refers both to the sense of position and sense of movement. These two components strongly contribute to fine motor control during voluntary movement execution, but are likely to be used in distinct cortical processes (Proske and Gandevia, 2009). Indeed, Dukelow et al. (2012) showed that it may be possible to discriminate deficits in position sensing from deficits due to the inappropriate utilization of afferent feedback in motor control.

Within this framework, we developed a proprioception-based motor training technique to augment kinesthetic awareness via haptic feedback mediated by a robotic manipulandum. Subjects have to perform targeted reaching movements in the absence of vision under the guidance of a minimally assistive pulsed force field applied to the hand. The pulsed nature of the guidance provides subjects with transient kinesthetic clues about their position relative to the target, inducing them to focus on their haptic sensation in order to produce a movement in the correct direction. We have recently applied this paradigm to neurologically intact subjects, in order to validate its effectiveness and test the correlation of an index related to proprioceptive acuity, namely the Active Contribution *(AC) index* with psychometric measures of kinesthetic sensitivity (De Santis et al., 2014a). The *AC index* demonstrated to be sensitive to changes in perceptual sensitivity that are dependent on the direction of the force field with respect to the arm configuration. Similarly, it can be exploited to quantify variations in proprioceptive anisotropies that are due to neurological deficits. This approach has several aspects of novelty. First, it allows to quantify kinesthetic acuity in a directional manner, avoiding a psychometric evaluation that is less specific, usually very time consuming and unpractical during a rehabilitation session. Second, the protocol warrants a uniform level of task difficulty over the span of reaching directions throughout the training session: the intensity of the haptic feedback is adaptively modulated according to the kinesthetic performance along different movement directions and the level of guidance can be automatically adjusted to match variations that may arise from adaptation or mental fatigue. Finally, it represents the first attempt to integrate active perceptual and motor training with an online quantitative evaluation of performance within the same exercise.

In this work, we investigate the applicability of the kinesthetic training protocol to a group of unilateral chronic stroke survivors with absent to severe level of proprioceptive deficit. With this purpose, we aim to characterize kinesthetic acuity of the stroke group when compared to healthy subjects. Psychometric parameters related to proprioceptive acuity in a two-choice force direction discrimination task of the healthy control group were used as a baseline reference for evaluating the effectiveness of the proposed protocol in enhancing sensation of the study group. Since the reaching paradigm involves the production of active movement, we are also interested in testing if any beneficial effects on the group motor performance are present as a consequence of the kinesthetic training.

## Materials and Methods

### Subjects

Seven right-handed stroke survivors (2M + 5F, 52.9 ± 14.0 years old) participated in this study. Table 1 reports their record and the clinical relevant data. The subjects were recruited among those followed as outpatients of the ART Education and Rehabilitation Center in Genoa according to the following inclusion criteria: (1) diagnosis of a single, unilateral stroke verified by brain imaging; (2) sufficient cognitive and language abilities to understand and follow instructions; (3) chronic condition (at least 1 year after stroke); (4) stable clinical conditions for at least 1 month before being enrolled in this study. Thirteen right-handed subjects with no previous history of neurological disease were used as reference group for proprioceptive acuity measures [for details, see De Santis et al. (2014a)]. Given the preliminary nature of the clinical study, we did not design the study to include a stroke control group that does not receive the robotic treatment. We assumed that simple familiarization with the device and exposure to the robotic assessment procedure over multiple days alone would not bring to significant perceptual alterations, since the subjects were involved in no concurrent therapy or physical exercise and their functional conditions were stable.

**Table 1:** **Record and clinical data of subjects**.

Subject	Age (years)	Stroke (years)	Gender (F/M)	Etiology (I/H)	Lesion site	Paretic hand	FMA (0–66)	MAS (0–4)	NAS (0–3)
S1	33	6	F	H	Sylvian and frontal area – RH	L	21	2	1 1 0 0
S2	64	9	F	I	Frontal and parietal area – RH	L	34	1	2 2 0 0
S3	39	10	F	I	Frontal and parietal area – RH	L	15	1 +	2 2 0 0
S4	65	15	F	H	Occipital area – RH	L	55	1	3 2 0 0
S5	66	12	M	I	Basal ganglia and internal capsula – RH	L	22	3	3 3 3 3
S6	43	4	M	H	Left superior capsular nucleus – LH	R	35	2	3 3 3 3
S7	60	6	F	I	Parietal area – LH	R	26	1 +	3 3 2 3

*Age is in years; Stroke: years after stroke; Gender: male (M) or female (F); Etiology: ischemia (I) or hemorrhage (H); Lesion site: stroke localization area(s) in the brain – RH = right hemisphere, LH = left hemisphere; Paretic hand: right (R) or left (L); FMA: Fugl-Meyer, Arm Section, 0–66 points; MAS: Modified Ashworth Scale, 0–4 points; NAS: Nottingham Assessment Scale, kinesthetic sensation portion, 0–3 points: [shoulder, elbow, wrist, hand]*.

The research conforms to the ethical standards laid down in the 1964 Declaration of Helsinki, which protects research subjects and was approved by the ethics committee of Regione Liguria. Each subject signed a consent form conforming to these guidelines. The robot training sessions were carried out at the Motor Learning and Robotic Rehabilitation Laboratory of the Istituto Italiano di Tecnologia (Genoa, Italy), under the supervision of experienced clinical personnel and engineers. All stroke subjects underwent clinical evaluations before starting the present study to ascertain their degree of spasticity [Modified Ashworth Scale – MAS (Bohannon and Smith, 1987)], residual functional level [arm portion of the Fugl-Meyer Scale – FMA (Fugl-Meyer et al., 1975)], and their proprioceptive deficits/impairments [kinesthetic sensation portion of the Nottingham Assessment Scale – NSA (Lincoln et al., 1998)].

### Protocol

**Figure 1:**
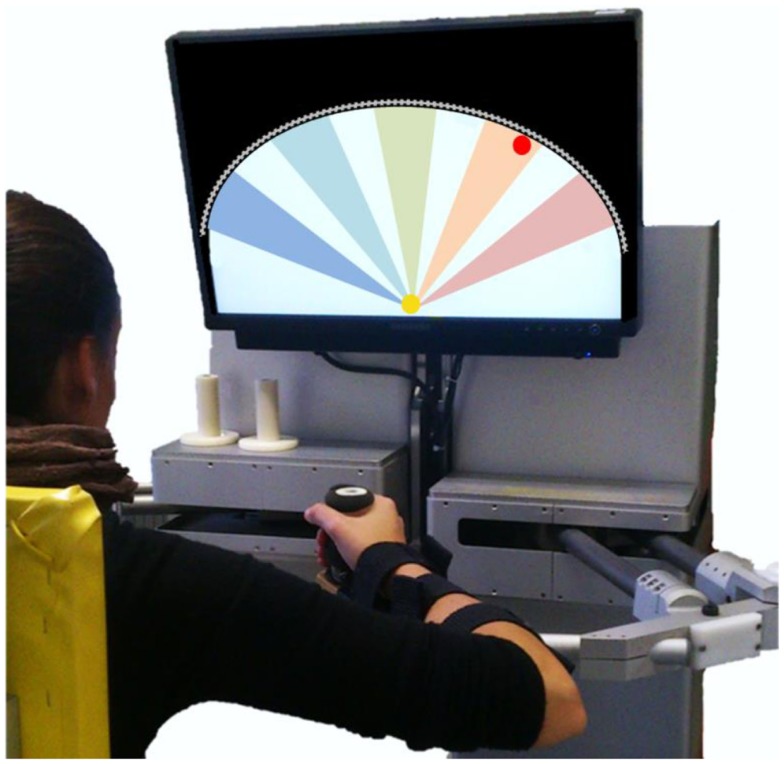
**Experimental setup**. The screen located in front of the subject showed the actual hand position (yellow circle, ∅ 2 cm) and the current target (red circle, ∅ 2 cm). The workspace is limited by a virtual wall (gray line) that subjects cannot overstep. The five colored sectors on the screen represent the five main reaching directions: −45°(blue), −22.5°(light blue), 0°(green), 22.5°(orange), 45°(pink).

Subjects sat comfortably on a chair in front of a robotic manipulandum (Casadio et al., 2006) that allows for shoulder and elbow movements along the transversal plane. To restrict trunk motion and to avoid compensation, the torso was strapped to the seat by belts. The seat position with respect to the manipulandum was adjusted in the frontal direction to allow for both shoulder and elbow flexion and extension movements throughout the workspace. The seat distance was set for subjects to reach the extreme point of the workspace with the full-extended arm, and a reference position 10 cm below the center of the workspace, with the elbow flexed by approximately 45°. Laterally, the seat was positioned to align the hand with the shoulder center and the workspace midline. The main task required subjects to hold the handle of the manipulandum with their most affected hand and complete a sequence of reaching movements. The forearm was upheld against gravity by a lightweight support connected to the handle of the robot. Vision was obscured throughout most of the experiment. When vision was required, an LCD screen placed in front of the subject provided real-time visual feedback of target and hand positions on the plane. Figure 1 shows the experimental setup.

The protocol comprised 8 sessions: 3 evaluation sessions (V1–V3) and 5 training sessions (T1–T5). Each session lasted approximately 1 h and subjects were allowed to take 5-min breaks whenever necessary. Subjects entrained for 5 days over 2 consecutive weeks. The evaluation blocks preceded and followed the training and were distributed as followed: an initial evaluation (V1) a few days prior to the first training session; a final evaluation after the last session of training (V2); a follow-up evaluation 1 week after the final evaluation session (V3).

Each *evaluation session* consisted of a psychometric estimate of the subject’s kinesthetic sensation on their most affected hand during a two-alternative forced choice discrimination test. The robot applied a sudden and quick force stimulus that displaced the arm randomly in two different directions (45° on the right or left with respect to the shoulder-elbow line during a full forward reach). Subjects, blindfolded, were asked to report the perceived direction of the arm displacement that occurred a few hundred milliseconds after an acoustic trigger. To enforce reliability before the beginning of a new trial, the robot repositioned the handle in the reference position and held it for 2 s. After this phase, the handle was released and the acoustic trigger was given only if the handle distance fell within 5 cm and the handle speed was less than 0.01 cm/s. The profile of the force stimulus was bell-shaped, with a duration of 200 ms and a peak value chosen pseudo-randomly in a user-defined force range according to a supervised Constant Stimuli approach. Prior to the beginning of the test, subjects underwent a familiarization phase to help them understanding the task correctly. After they got accustomed with the task, a first set of 10–20 grossly spaced stimuli (0.5–1 N) was used to identify the range of peak impulse amplitude that allowed for a coarse psychometric estimate. Thereafter, the stimulus intensity range and grain was adjusted to sample the range of stimuli that yielded to a probability of positive response greater than 60%. This procedure was repeated until increasing the number of trials did not affect significantly the 85% probability of positive response threshold estimate (variation < 0.1N).

The *training sessions* were divided into two separate blocks. Each session begun with a shortened psychometric evaluation to tune the level of haptic guidance to be used in the subsequent exercise block. The initial haptic guidance level was chosen equal to the 85% probability of correct discrimination. The exercise block consisted of a reaching task: subjects had to reach a set of 45 target points distributed along 5 evenly spaced circular sectors of 10° of amplitude centered in a starting position, as shown in Figure 1. To prevent subjects from memorizing the target locations, we introduced 5° of variability in their position so that each of them could pertain to the center (0°, ±22.5°, ±45°) or to the extremes (center ± 5°) of a sector, yielding 15 target positions in total. The distance to be covered from the starting position was modified according to subjects’ degree of spasticity: 12 cm if MAS > 1, 15 cm otherwise. The sequence of target presentations alternated the starting position and one of the peripheral targets in a pseudo-randomized order. A target set included 3 reaching movements for each peripheral target, for a total of 45 center-out movements, plus 45 return movements. An auditory feedback signaled that a target was hit (handle distance to the target equal to 2 cm). In each training session, subjects performed at least two target sets, the first having both visual and force feedback (VFF) and the subsequent target sets having FF only. In the VFF condition, subjects were instructed to reach the displayed target moving as straight as possible, but with no timing or speed constraints. The color of the target ball was turned from red to green whenever the straightness along a direction improved. Straightness was quantified by computing the *AC index* online (see Outcome measures). In this first condition, subjects were not informed about the presence of an assistive force acting throughout the target set. On the contrary, in the FF only condition subjects were instructed that the reaching task had to be performed without any visual feedback and under the light guidance of the robot. To inform them on where to move to, the robot would have provided them with gentle pushes similar to the one they experienced in the proprioceptive test. Subjects were asked to focus on the direction of the robot’s movement and move their arm along with it until they heard a sound. The number of target sets in the FF condition could vary depending on the subjects’ level of physical or mental fatigue.

As part of the first evaluation session, subjects were allowed to familiarize with the training task performing a target set with both feedback conditions.

### Force feedback

The haptic feedback consisted of a series of force impulses, directed as the line joining the hand position and the target as described in Eqs 1 and 2 in Supplementary Material. The impulse duration was fixed to 200 ms and the frequency of the train of impulses to 2 Hz. A continuous bias force was added to the impulse train in order to help impaired subjects to complete the requested task. In addition, a viscous field mitigated the elastic back-bounce due to the impulse application and a virtual wall acted as a haptic elastic barrier for the hand 2 cm beyond the target distance.

The level of continuous force to be applied in the VFF condition was initially evaluated for each subject during the familiarization target sets (V1) according to their degree of spasticity as assessed by the MAS between 1 and 2 N [*F*_A_ = 0.5 N (MAS = 1); *F*_A_ = 1 N (MAS = 1 +), *F*_A_ = 1.5 N (MAS = 2), *F*_A_ = 2 N (MAS = 3)]. When visual feedback was absent (FF condition), we found that a much lower continuous assistance was required. Therefore, for a given subject, the *F*_A_ level was reduced up to 0.5 N and anyway set to be inferior to the estimated psychometric threshold.

The initial level of pulsed guidance *P*(*t*) was chosen according to the psychometric discrimination curve as the stimulus intensity that yielded to a probability of positive response equal to 85% (*F*_85_). The peak pulsed assistance value was kept constant throughout the trials in the VFF condition. Conversely, in the FF condition, the peak amplitude was adaptively modulated according to the proprioceptive performance as estimated by the *AC index*.

### Outcome measures

**Figure 2:**
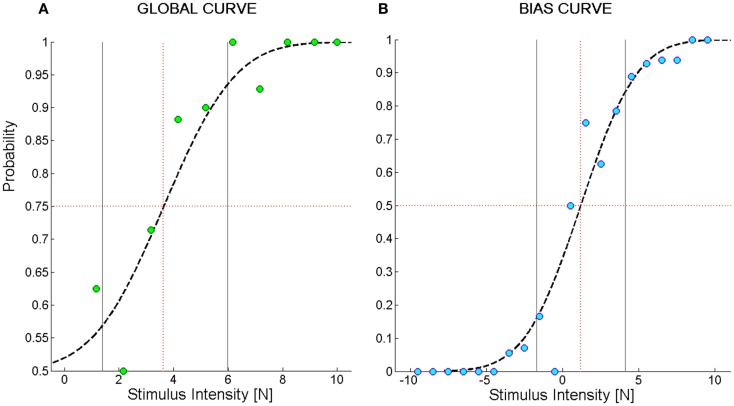
**Example of psychometric curves from the force direction discrimination task**. **(A)** *Global Curve*; **(B)** *Bias Curve*. The ordinate values indicate the probability of perceiving the direction of the force stimulus; the abscissa represents the magnitude of the force stimulus. In **(B)**, negative stimuli stand for forces directed to the left and positive stimuli for forces directed to the right. The green and blue filled circles represent the average probability to perceive the stimulus in the correct direction over bins of 1N. Subject’s responses were fit to a logistic function (dashed black line). The black vertical lines represent the interval of stimuli one standard deviation far from the mean of the Gaussian probability function used to fit the data. We called this interval *Spread*. The red dotted vertical lines identify the stimulus corresponding to its mean value that we named *F*_85_ for **(A)** and *Bias Level* for **(B)**.

To characterize the kinesthetic sensitivity of each subject to a directed force impulse in the two-alternatives forced choice discrimination task, a cumulative Gaussian function was fitted to the dataset and two types of psychometric sensitivity functions were derived. In one case (Figure 2A), we computed the percentage of correctly perceived force stimuli independent of their direction. The fitted logistic function is limited to the interval [0.5–1] and we called it *Global Curve*. In the other case (Figure 2B), we took into account the stimulus direction by computing the probability of perceiving a perturbation as rightward given a stimulus directed 45° to the left or 45° to the right. We called the fitted logistic function *Bias Curve*. It ranges between 0 and 1, with leftward stimuli represented as negative forces.

To summarize the information represented in the *Global Curve* and the *Bias Curve*, the following set of indicators were adopted:

1. *F*_thr_ [N]: represents the minimum force that allows for identifying the direction of a stimulus. It is equal to the force intensity yielding a probability of giving a correct answer equal to 75%. It is computed on the *Global Curve*.
2. *F*_85_ [N]: reflects the acuity of the subject in the discrimination task. It is equal to the force intensity yielding a probability of giving a correct answer equal to 85% and is computed on the *Global Curve*.
3. *Spread* [N]: is a measure of the uncertainty of subjects in identifying the direction of the force. It is computed as the standard deviation of the Gaussian probability function corresponding to the *Global Curve*.
4. *Bias level* [N]: is the stimulus intensity corresponding to a 50% probability of perceiving a stimulus as directed toward the right. It is computed on the *Bias Curve*. A negative bias level indicates that, for a given intensity, stimuli directed to the right are perceived more acutely than stimuli directed to the left. This is because, for instance, the intensity required to elicit a sensation that is 75% the times correct is higher when a stimulus displaces the hand to the left than to the right.

In order to quantify the kinesthetic performance of the subjects and their ability to exploit the proprioceptive feedback to guide targeted reaching movements, the following two indicators were adopted:

1. *Trajectory Shift (Shift)* [cm]: it is computed as the mean lateral deviation from a linear trajectory since the movement initiation until the target is reached. The onset of movement is computed as the first instant after the new target presentation in which the speed exceeds a threshold of 0.01 m/s.
2. *AC index*: it measures the appropriateness of the motor response to the pulsed force stimulus. It is computed, as described in detail in Supplementary Material, by taking into account the relationship between the impulse train and the corresponding kinematic modifications of the trajectory. Its value ranges between 0 and 1.

### Assistance modulation

The goal of the algorithm, which was originally described in De Santis et al. (2014b), is to identify and track in time the minimum force level *F*_PEAK_ that allows for a desired kinesthetic performance in a reaching movement along a specific direction. The *AC index* is used here as a measure of kinesthetic proficiency and the desired performance.

The desired level of kinesthetic acuity was selected independently for every subject. Since the *AC index* measure could be influenced by the individual motor performance in the reaching task, we computed the average *AC* values in each direction sector obtained by a subject during the target set when also visual feedback was provided. Hence, the desired kinesthetic performance was set equal to the 85% of the average *AC* score of the subject in the VFF condition. This choice allowed the algorithm to account for the presence of possible motor deficits.

As explained in Supplementary Material, which also addresses the issue of stability of the algorithm, the force level in a trial is adaptively regulated to minimize the distance from the desired performance: whenever the performance is insufficient, the force is increased; if the performance is superior to the desired level, the force is decreased.

### Data analysis

Data analysis is structured by taking into account the two main objectives of this study: (i) characterizing kinesthetic acuity in unilateral stroke survivors compared to healthy subjects and (ii) evaluating the applicability and effectiveness of a new protocol for kinesthetic training. For this purpose, we first compared the psychometric parameters of the stroke subjects (*F*_thr_, *F*_85_, *Spread*, and *Bias level*) before (V1), during (T1–T5), and after the training at different time instants (V2, V3) with the values obtained for the healthy control group. In particular, we hypothesized that the psychometric estimate of the assistive force that subjects requires to carry out the reaching task, namely *F*_85_, will decrease as a consequence of the exercise. To test this hypothesis, we adopted a Friedman’s ANOVA for dependent samples of *F*_85_ over the training sessions. The test was repeated over the three evaluation sessions in order to inspect the subject’s ability to retain possible improvements on the perceptual score. Effects were considered significant if exceeding the α = 0.05 threshold. A *post hoc* analysis was then used to identify the significant comparisons.

Second, we wanted to examine if changes in the *F*_85_ level were reflected by an increased/decreased ability of the subjects to employ the kinesthetic information during the reaching task. Therefore, we computed the average level of force required for the subjects to achieve the target performance level of *AC index* = 0.85 (−5%) along the five sectors (−45°, −22.5°, 0°, 22.5°, 45°) and we compared the values at the first (T1) and the last training session (T5). To limit the influence of fatigue and of the settling time of the assistance regulation algorithm, only the force intensities in the middle trials were considered. In order to compare the *AC index* among subjects, the values were corrected for the difference between the individual desired performance *AC*_d_ and the ideal value of 0.85.

Finally, aiming to test if the kinesthetic practice was beneficial to the overall movement performance, we computed the mean absolute value of the *Shift* indicator for the trials in which visual in addition to haptic feedback was provided (VFF condition). We compared the mean value of the indicator over subjects in the five main directions in the first with respect to the last training session. In order to account for the differences in the limb configuration among right-affected and left-affected individuals, we mirrored the values of the *F*_85_ and *Shift* parameters computed for different directions with respect to the midline of the workspace.

## Results

### Psychometric parameters

**Figure 3:**
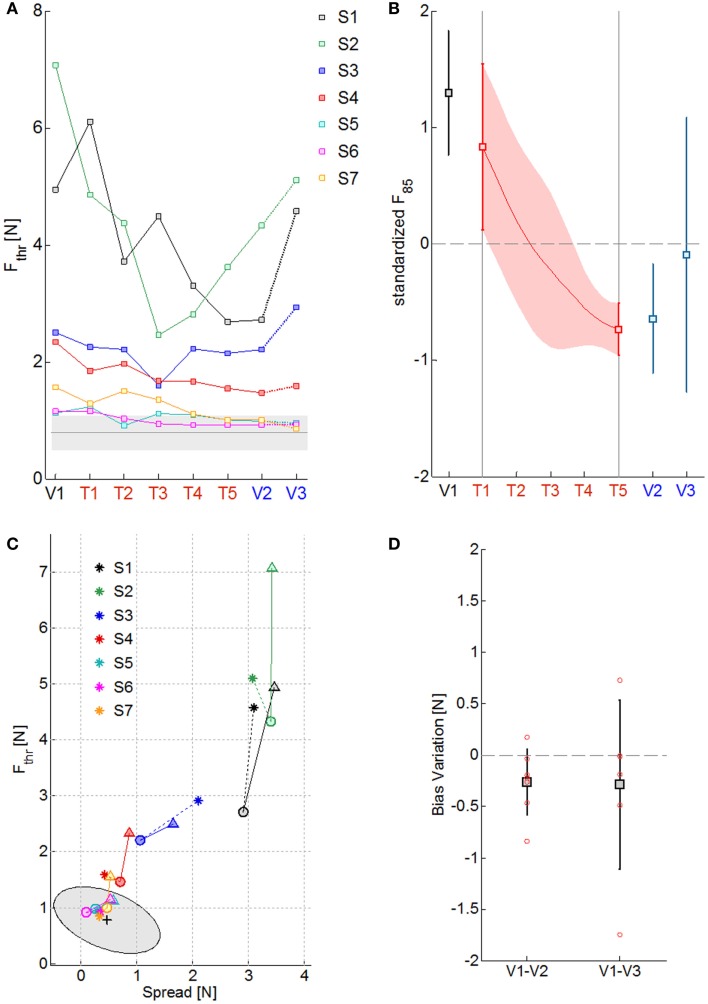
**Summary of the psychometric measures of kinesthetic acuity over sessions**. **(A)** *F*_thr_ values along the evaluation (V1–V3) and training sessions (T1–T5) for the seven subjects, identified with different colors. The gray line is the mean *F*_thr_ value obtained for the healthy control group and the shaded area the corresponding standard deviation. **(B)** Standardized *F*_85_ values over subjects in the 8 sessions (squares and continuous red line) and their corresponding standard deviation (vertical lines and red shaded area). **(C)** Effect of training over the psychometric sensitivity (*F*_thr_) and the response uncertainty (*Spread*): triangles, circles, and asterisks indicate measures obtained respectively at V1, V2, and V3; colors represent different subjects; the mean performance of healthy control subjects is rendered by the black cross and the gray ellipse region represents the ellipse of variation along the principal components of the distribution. **(D)** Variation in the absolute value of the *Bias level* from the first to the final evaluation session (V1–V2) and to the follow-up evaluation session (V1–V3): squares stand for the mean variation, the total length of the error bars represent two standard deviations; single subject data are represented by the red circles.

The four panels of Figure 3 summarize the psychometric indicators in different phases of evaluation and training. Figure 3A reports the level of the sensitivity to the force displacement direction for the seven impaired subjects (colored lines) compared to the average value of the neurologically intact group (gray line) throughout the sessions. As it can be noted, the stroke subjects greatly differ on their psychometric threshold in the direction discrimination task. In particular, three groups of subjects can be identified, based on the indicator value: severe kinesthetic impairment (S1 and S2), moderate impairment (S3 and S4), mild or absent impairment (S7, S8, and S9). As a consequence of training, all the subjects tend to decrease their psychometric threshold with respect to the initial evaluation, but only the subjects with a mild level of impairment converge to the normality range. To seek for a significant effect of training on the proprioceptive sensation, the psychometric data of each subject were detrended and normalized for the corresponding standard deviation over sessions. Figure 3B depicts the average standardized *F*_85_ value and its variability in time. The statistical test found a significant effect of the exercise on the *F*_85_ values over training [χ^2^ (*N* = 7, *df* = 4) = 16.08, *p* = 0.0029] starting from the fourth training session (T4). The same test repeated on the evaluation sessions highlighted that there is a carry-over effect immediately after the training (V2) but that the subjects are generally unable to retain the improvements after a week (V3) [χ^2^ (*N* = 7, *df* = 2) = 8.00, *p* = 0.0183]. Since the *F*_85_ parameter is dependent both on the threshold level and on the steepness of the cumulated probability distribution, one may wonder if both aspects contributed equally to the enhancement and subsequent decay of the improvements with the time after training. Figure 3C extrapolates the information related to the uncertainty in the stimulus identification and compares it to the discrimination threshold over the evaluation sessions (V1 = triangle, V2 = circle, V3 = asterisk) for every subject. Apparently, there is no homogeneous behavior among the most severely impaired subjects that fail to retain the gains in both parameters. However, the best performing subjects (S4–S7) are in general better at preserving the sensitivity information rather than the discrimination accuracy. As far as the *Bias level* parameter is concerned, at V1 all the subjects presented uneven perception of rightward and leftward stimuli (absolute bias on average at V1: 0.54 ± 0.58 N). Nevertheless, as Figure 3D shows, repetitive practice with our protocol helped the subject to improve the *Bias level* after the training (V2: 0.27 ± 0.33 N, V3: 0.30 ± 0.32 N), despite the values being still higher than the control group ones (0.18 ± 0.1 N for the right arm and 0.14 ± 0.09 N for the left).

### Force feedback

**Figure 4:**
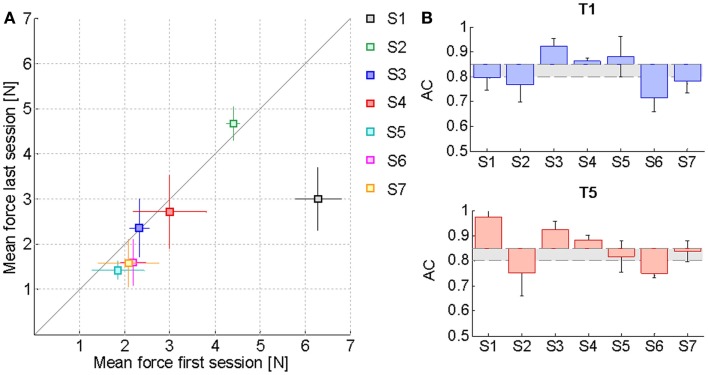
**Effect of training on the assistive force**. **(A)** Mean *F*_PEAK_ values and corresponding standard deviation over the middle trials of the first training session compared to the last one. The solid line represents the equality condition. Points that lie below the line indicate that the average force needed by the subjects for carrying out the reaching task has diminished, while points above the line denote an increase in the average haptic guidance. **(B)** Mean *AC index* variation with respect to the desired level of kinesthetic performance *AC*_d_ = 0.85 in the first training session (T1) and in the last one (T5). The gray area includes all the AC values within the tolerance margin of 5% allowed below the desired AC percentage. Values have been corrected for the difference between the actual *AC*_d_ of each subject along a specific direction and the ideal value of 0.85.

Table 2 reports the *F*_85_ values estimated from the psychometric evaluation along the sessions. In the preceding section, it was remarked that this parameter decreases significantly with kinesthetic practice. We hypothesized that a reduction in the estimated initial level of assistance would have been reflected by an increased ability of the subjects to employ the kinesthetic information in the reaching task. Figure 4A shows the effect of training on the assistive force that the assistance regulation algorithm estimated for the subjects given their kinesthetic performance according to the *AC index*. The mean *AC* level corresponding to the selected trials in the first (T1) and the last (T5) training session is also shown in Figure 4B. The *AC* values are in general within or above the desired threshold, indicating that the force estimate we considered is conservative, since subject could potentially achieve the target performance with lower forces. Despite the difference being not statistically significant, all the subjects but two (S2, S3) succeeded in reducing the amount of guidance required for carrying out the kinesthetic reaching. In the case of S3, however, the level of guidance did not increase significantly on average and the kinesthetic score is above the minimum threshold.

**Table 2:** **Estimated pulsed assistance level through sessions and subjects**.

Subject	*F*_85_ [N]							
	V1	T1	T2	T3	T4	T5	V2	V3
S1	6.75	6.00	5.76	6.18	4.65	4.04	4.24	6.20
S2	8.00	5.76	5.07	2.51	4.44	4.37	6.11	6.71
S3	3.37	3.10	3.09	2.61	2.57	2.62	2.77	4.02
S4	2.79	2.36	2.27	2.36	1.99	1.94	1.83	1.81
S5	1.43	1.52	1.10	1.28	1.22	1.13	1.12	1.10
S6	1.43	1.35	1.18	1.05	1.03	0.97	0.97	1.11
S7	1.84	1.56	1.83	1.56	1.29	1.25	1.25	1.03

### Motor performance

**Figure 5:**
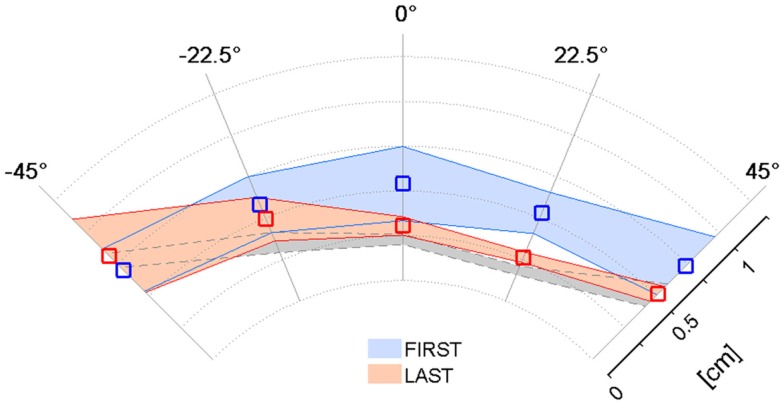
**Effect of training on the Shift parameter in the VFF condition**. Mean absolute S*hift* values and corresponding standard deviation over the target set with both visual and force feedback on the first (blue) and last (red) day of the kinesthetic training; the gray shaded area corresponds to the performance of the healthy control group.

The previous results relative to the psychometric parameters and the assistive force highlighted that the kinesthetic practice was on average beneficial in the sense that strengthened the subjects’ ability to employ the FF in targeted reaching movements in the absence of visual feedback. Since the kinesthetic feedback contributes to the overall motor performance, we examined if the enhanced kinesthetic acuity had any influence on the reaching task when visual information about the hand and target position was added. Figure 5 compares the mean absolute *Shift* values in the five direction sectors computed in the first (blue) and the last (red) training sessions. The average reference performance of the neurologically intact group is depicted in gray. In the first target set, stroke subjects performed much less accurate reaching movements than the control group (0.52 ± 0.3 cm), yielding an average trajectory shift of 0.62 ± 0.1 cm. In contrast, after the practice, the mean absolute shift improved more than the 20% (0.48 ± 0.2 cm), despite failing to reach significance in a *t*-test comparison [*t*(34) = 1.77, *p* = 0.085], and its values over the five directions closely match the control group performance. We report that the greater variability observed on the movement directions that require arm and shoulder extension is mainly due to the onset of spasticity in S5.

## Discussion

Three main findings can be singled out in this work, focused on kinesthetic evaluation and training: (i) the proposed protocol is able to enhance kinesthetic awareness in chronic stroke subjects, as evaluated by the psychometric test; (ii) repetitive training with directional force pulses led subjects to a more efficient use of the kinesthetic feedback in guiding targeted reaching movements; (iii) the ability of subjects to retain the functional improvements is negatively correlated with the level of proprioceptive impairment. In this section, we further discuss the reliability of psychometric measures, the effects of training, and speculate about the plausible influence of the proprioceptive deficits on plasticity. Finally, we will address some of the possible limitation of the study and suggest further improvements.

### Reliability of psychometric measures of proprioception

It is generally accepted that proprioceptive information is essential for the correct calibration of motor commands (Ghez et al., 1995) and the control of limb posture. Somatosensory deficits are a very common outcome after stroke and may impact severely on the ability of subjects to recover function of the affected limb as well as increase the risk of further injuries. Therefore, assessment is a first step toward the identification and correct treatment of proprioceptive deficits. In order to cope with the poor reliability and the coarseness of clinical assessments, quantitative tests for proprioception are needed (Carey et al., 1993; Simo et al., 2014). In our work, kinesthetic sensation was evaluated by using a psychometric procedure. The affected arm of the subject was suddenly and briefly displaced in either a 45° right or left direction by a robotic device imposing directed forces of variable intensity to the hand. The kinesthetic sensation was quantified through the ability of subjects to discriminate between the two possible directions of displacement at different force magnitudes. This method is alternative to the most widely used arm position matching test, in which subjects have to actively match with the affected limb the position of the hand of a reference limb (affected itself or healthy) in space following its passive placement by a robotic device in the absence of visual feedback (Goble and Brown, 2007; Dukelow et al., 2010; Semrau et al., 2013). The same test can be performed by requiring the subject to match the configuration of the joints of a reference arm (Leibowitz et al., 2008). However, the two tests are not equivalent both in terms of cognitive requirements and results (Elangovan et al., 2014; Iandolo et al., 2014) that are usually conditioned by the involvement of memory, inter-hemispheric transfer of proprioceptive information, and the need for active subject movements that may be problematic after stroke. For these reasons, Elangovan et al. (2014) suggested the use of psychometric estimates of kinesthetic detection threshold of the elbow in a passive movement discrimination test as a method to improve reliability and reduce variability in position sense testing. On the other hand, Simo et al. (2014) developed a psychometric detection test for quantifying the sensitivity to controlled arm movements and to sinusoidal force perturbations. Based on the results, they were able to identify a maximum likelihood boundary that allows for discriminating with high reliability between intact and impaired proprioception at the arm. The force direction discrimination test adopted in the present experiment may be conceived as a further step in this direction because it extends the evaluation of the position sense with the correlated functionality of the movement sense. The results of the two experiments are indeed complementary. The shape of the variability ellipse we used to fit the non-impaired subject scores closely matches the maximum likelihood boundary region identified in the detection tasks. Moreover, when considering the relationship between the force direction discrimination threshold and the related stimulus uncertainty, the level of kinesthetic impairment appears to be related to the distance from the healthy control subjects’ distribution. Proprioceptive dysfunctions after stroke are, therefore, characterized by higher discrimination thresholds and reduced confidence in the identification of the direction given the force stimulus. The threshold values and uncertainty range detected by Simo et al. (2014) were in general lower than the ones we found. This difference may be accounted for by the higher complexity of the task (discriminating the stimulus direction other than the simple presence of a hand displacement). However, the kinesthetic perturbation adopted in the perceptual test was compatible with the sensorimotor challenge required by the training exercise. The fact that the results obtained are strictly comparable in these two works, strengthen the reliability of evaluating proprioceptive deficits through psychometric correlates of kinesthesia.

### Effect of training

As a result of repetitive practice with the kinesthetic training protocol, we have shown that subjects are able to minimize the intensity of the FF – and therefore the magnitude of imposed arm displacements – that is necessary for them to reach a comparable kinesthetic performance in time. This improvement can be accounted for by several factors. Two critical features of the proposed training protocol are its attentional demand and in particular the attentional target. In both the evaluation and the training sessions, subjects were required to constantly focus on their affected limb in order to detect not only its motion but also in particular the direction of the imposed hand force. It is known that spatial and non-spatial attentions are frequently impaired after stroke, in particular in the presence of neglect (Corbetta and Shulman, 2011). Patients generally exhibit a rightward bias in perception that has been shown to be affected by modulation of attention (Van Vleet et al., 2011) and passive movements of the left limb (Frassinetti et al., 2001). On the other side, active limb movements seem to be critical for improvement (Robertson and North, 1993). What is of key importance is that the magnitude of the improvement is strictly related to the saliency of the proprioceptive information. If meaningful, limb activation has the potential to modulate, at least transiently, the sensorimotor representation of the limb known as body schema (Reinhart et al., 2012), which is specifically used to guide action (de Vignemont, 2010). Therefore, we argue that the effectiveness of a training protocol for proprioception resides in its ability to recruit voluntary, target-oriented movements in conjunction with meaningful feedback information. The protocol adopted in this work was specifically designed to enhance kinesthetic awareness through repetitive stimulation of afferent feedback through transient haptic interaction. Not only have the subjects to perceive and identify an imposed arm perturbation, but they especially need to correctly interpret and exploit the feedback information to produce a target-oriented movement. Since the haptic clue is discontinuous, this process has to take place incrementally, forcing the subject to constantly elicit the action-perception network. This, in time, may lead to a recalibration of the internal models of the affected limb. In their studies on deafferented subjects, Gordon et al. (1995) suggested that not only does the proprioceptive input contribute to the control of movement by providing feedback for corrections but also it directly affects human ability to form and update internal representations of the biomechanical properties of the limb. These observations may relate to our finding that subjects improved the linearity of reaching movements with visual feedback after kinesthetic training. Such result may be due as well to an increased attention or consciousness about own limb’s movements. Since the visual feedback has not changed, we may hypothesize that the uncertainty related to the proprioceptive feedback decreased with training, leading to a better integration of feedback information (Scheidt et al., 2010). Anyway, even though subjects mostly practiced without visual feedback, we cannot exclude that trials in which both feedback channels were present could improve performance.

### Influence of the proprioceptive deficits on plasticity

The present study demonstrated that kinesthetic training can consistently modulate perception in stroke survivors. However, the modulatory influence of the haptic exercise appeared to be only temporary in three out of seven subjects: they failed to retain the proprioceptive improvement after 1 week. Interestingly, this reversal effect occurs only for subjects with moderate to severe impairment in kinesthesia (S1–S3). On the other hand, less impaired subjects demonstrate to further improve their perceptual scores 1 week after the end of the training. This result should not be surprising, since a number of earlier studies found that the severity of perceptual deficits negatively impact on the chance of recovery (Kusoffsky et al., 1982; Smith et al., 1983; Rand et al., 1999; Schabrun and Hillier, 2009). Han et al. (2008) suggested that the efficacy of rehabilitation treatments depends on their ability to increase spontaneous arm use. Hence, it is possible that either the intensity of the proposed protocol alone was insufficient to promote durable changes in the somatosensory cortex of the most impaired subjects or the spontaneous use of the arm in daily activities was anyway limited by their low residual functional level. However, a recent study by Vahdat et al. (2014) argued that perceptual learning may directly contribute to persistent changes to motor areas of the brain directly related to motor learning. We believe that the proposed kinesthetic training, that comprises in itself both motor and perceptual aspects, may greatly benefit from the integration with other forms of motor therapy. In this way, it is possible to promote and support the execution of active movements to bring to a functional re-learning by use that opposes to the learned non-use that frequently occurs after hemiparesis.

### Limitations of the study and implications for future work

In the present work, we validated a new procedure for kinesthetic training based on an adaptive regulation of assistance to enhance limb awareness in chronic stroke survivors. Session after session, a threshold difficulty value is selected according to the subject’s performance during the open eyes condition. Our results highlighted that subjects improved in their movement accuracy and significantly on proprioceptive discrimination acuity throughout the training. Notwithstanding the positive results, it should be noted that the presented experimental protocol has a number of limitations. Given the exploratory nature of this study, the number of subjects we tested was small, presenting different degree of motor and proprioceptive impairments to investigate the impact of different functional deficits on the training efficacy. We found in particular that the severity of proprioceptive impairment hinders long-term adaptation. Additional studies would be needed, involving a greater number of stroke subjects and a longer training period to inquire into the dynamics of recovery as a function of the degree of the proprioceptive loss. Furthermore, the assessment phase would benefit from the integration with additional proprioceptive measures (i.e., arm matching/position matching task) or motor tasks (i.e., free movements) to test for any generalization effect due to the kinesthetic training.

A second consideration should be made on the role of the assistance regulation algorithm. The proposed procedure was designed to stabilize the performance of healthy subjects around a chosen threshold within 90 trials (two target sets), given a range of admissible guiding forces. Therefore, we can outline three levels of arbitrariness that could possibly influence the learning process: the choice of the force range, the choice of the initial force step involved in the update of the assistance level from trial to trial, and the choice of the target kinesthetic performance. To limit the variability, in the present experiment, the force range and the initial step were chosen according to the psychometric evaluation to be consistent with the uncertainty of the response and the range of perceivable stimuli. As for the choice of the target performance, we set it equal to 85% of the visually guided trials. Under the hypothesis of rectilinear motion, this value corresponds to an angular deviation of approximately ±10° with respect to the target direction that is the average distance between two adjacent target points. However, we observed that subjects with pronounced spasticity tended to perform much better when the visual feedback was removed compared to the vision condition. In this case, the computed target level would correspond to a lower challenge condition, and possibly limit the improvement. In order to account for the misbalance, the algorithm should be able to update the reference performance level increasing the accuracy requirements whenever reaching a threshold.

## Conflict of Interest Statement

The authors declare that the research was conducted in the absence of any commercial or financial relationships that could be construed as a potential conflict of interest.

## Supplementary Material

The Supplementary Material for this article can be found online at http://www.frontiersin.org/Journal/10.3389/fnhum.2014.01037/abstract
