# Supplementary material for: Robot-Assisted Training of the Kinesthetic Sense: Enhancing Proprioception after Stroke
Source: Front Hum Neurosci. 2015 Jan 5;8:1037. doi: 10.3389/fnhum.2014.01037 (PMC4283673; doi:10.3389/fnhum.2014.01037)
Supplement: Supplementary file 1 [file Presentation_1.PDF]

Appendix

Force feedback

The haptic feedback consisted of a series of force impulses $P(t)$ of amplitude F_{PEAK} and directed as the line joining the hand position x_H and the target x_T, as described in Equations (1) and (2).

$$P(t) = [F_{PEAK}.I_{\Delta t}(t)]\frac{(x_T - x_H)}{\|x_T - x_H\|} \qquad (1)$$

$$\xi = t/\Delta t$$

$$I_{\Delta t}(t) = \begin{cases} \frac{1}{1.875}[30\xi^4 - 60\xi^3 + 30\xi^2] & \text{for } 0 \le \xi < 1 \\ 0 & \text{for } 1 \le \xi < T/\Delta t \end{cases} \qquad (2)$$

The single impulse duration Δt was fixed to 200 ms and the frequency of the train of impulses to 2 Hz. A continuous force field F_A directed to the target was added to help subjects to complete the requested task. In addition, a viscous field mitigated the elastic back-bounce due to the impulse application and a virtual wall acted as a haptic elastic barrier for the hand 2 cm beyond the target distance. The net force field acting on the hand is described by Equation (3), where F_A is the continuous force bias, $P(t)$ is the pulsed haptic guidance force field, always directed towards the target point, B is the coefficient of the viscous field component (12 Ns/m), K_w is the elastic coefficient of the virtual wall (1000 N/m), x_H the position vector of the hand and x_w its projection onto the wall surface.

$$F(t) = F_A + P(t) - B\dot{x}_H - K_W(x_W - x_H) \qquad (3)$$

AC index

It estimates kinesthetic accuracy by measuring the appropriateness of the motor response to the pulsed force stimulus. For a given impulse cycle *i* (from one rising phase to the next one, *M* samples) we computed the net force vector over the cycle $\vec{F}_i = \sum_{j=1}^{M}\|\vec{P}_j\|$ and a partial AC_i index as the ratio between the movement length over the cycle and the line joining the position of the hand at the beginning and at the end of the cycle, as in Equation (4). The *AC* index is then computed in Equation (5) as the sum of the partial indicators over the *N* impulses weighted by the partial movement length S_i and penalized by the cosine between the net force $\vec{F}_i$ and the net hand velocity, α_i.

$$AC_i = \frac{\|\sum_{j=1}^{M}\vec{v}_j \cdot \delta t_j\|}{\sum_{j=1}^{M}\|\vec{v}_j \cdot \delta t_j\|} = \frac{\|\sum_{j=1}^{M}\vec{v}_j\|}{S_i}, \qquad S_i = \sum_{j=1}^{M}\|\vec{v}_j\| \qquad (4)$$

$$AC = \frac{\sum_{i=1}^{N}\alpha_i S_i \cdot AC_i}{\sum_{i=1}^{N} S_i}, \qquad \alpha_i = \frac{\langle\sum_{j=1}^{M}\vec{v}_j, \vec{F}_i\rangle}{\|\vec{F}_i\|\|\sum_{j=1}^{M}\vec{v}_j\|} \qquad (5)$$

where $\vec{v}_j$ is the instantaneous velocity vector at the jth sampled time instant, δt_j the infinitesimal time difference between the jth time instant and the (j-1)th, $\langle\cdot,\cdot\rangle$ is the inner product operator. Since the off phase of the impulse (300 ms) is greater than its active phase (200 ms), the contribution to the i-th score of the active movement executed in the absence of force is weighted more than the

movement executed during the impulse active phase. Whenever the subject is moving in the exact direction of the force along a linear path the AC index is maximum and equal to 1. On the contrary, the AC index is close to 0 either when the subject is not producing any active focal motor command in response to a force impulse (due to the bell-shaped impulse the net movement is close to zero) or when the movement occurs in a direction rotated more than 90° respect to the net force vector. Therefore, the higher the accuracy in perceiving the force and producing a movement in that very same direction, the higher the AC index.

Assistance modulation

The assistance modulation algorithm was developed to adaptively regulate the level of the guiding force F_{PEAK} to allow for a desired kinesthetic performance AC_d in a reaching movement along a specific direction. The most straightforward way to modulate the assistance level is to update the force intensity in a trial *k* proportionally to the error on the *AC* index, namely AC_d -AC^k, as follows:

$$F_{PEAK}{}^{k+1} = F_{PEAK}{}^{k} + \mu \Delta F^k \quad (6)$$

$$\Delta F^k := \begin{cases} \delta \cdot (AC_d - AC), & if\ AC^k < AC_d - \varepsilon \\ -\delta \cdot (AC_d - AC), & if\ AC^k > AC_d \\ 0, & otherwise \end{cases} \quad (7)$$

where μ [N] is the step size parameter and represents the gain of the corrective action on the actual value of the assistance; ε = 5% is the tolerance level below the desired performance AC_d for which we consider the performance ideal; δ = 10% is the fixed gain over the *AC* error. In this way, the force level is regulated to minimize the distance from the desired performance: whenever the performance is insufficient, the force is increased; if the performance is superior to the desired level, the force is decreased. The role of the tolerance margin ε is to reduce the sensitivity of the algorithm to fluctuations due to trial-to-trial variability in the *AC* measure.

The time required for the algorithm to stabilize the stimulus value is critical, and it is necessarily limited by the duration of the training session. Moreover, since a certain amount of adaptation in time might occur (Darainy et al., 2013), the algorithm should be able to respond promptly to changes in kinesthetic acuity in the course of the exercise. Given a reasonable initial force value $F^0 = F_{85}$,the speed of convergence of the algorithm to the desired performance is dependent on the choice of the step size parameter μ. To comply with the time constraints and the need for adaptation in time, we applied a reward-based learning procedure that modulates the step size throughout trials to balance exploration and convergence. For instance, at the beginning of the training session, the step size should be high enough to allow for the algorithm to rapidly move in the direction of the threshold. When the threshold is approached, instead, the step should be reduced to track the small modifications in the sensitivity to the force. In this way, the algorithm can keep track of the behavior through time and increase or decrease the step size according to how rewarding this action is. In particular, the maximum reward, equal to 1, was assigned to trials in which the selected force brought to an increment in the kinesthetic performance. The minimum reward was given to forces that caused a performance decrease. Equation (8) describes the reward assignment policy:

$$r^k := \begin{cases} 1, & if\ AC^k > \overline{AC}^n \\ -0.5, & if\ AC^k < (1-\varepsilon) \cdot \overline{AC}^n \\ 0, & otherwise \end{cases} \quad (8)$$

The behavior of the algorithm is monitored over a finite time window of $m = 3$ trials. After m trials, the algorithm computes the mean kinesthetic performance $\overline{AC}^n$ and the mean reward $\bar{r}^n$ over the observation window and takes a decision over the steps size following a Q-learning strategy with discount factor γ. After having observed the performance for the window n of m trials, the algorithm can take two possible actions: increase or decrease the step size. The rewards modify the probability $P(\cdot)$ of incrementing the step size (action a) as in equation (9), where α = 0.3 corresponds to the learning rate and expresses how much the rewards affect the current value; γ = 0.5 is a discount factor that weighs the influence of future vs current rewards:

$$P(a) = (1-\alpha)P(a) + \alpha\left(r^n \cdot \gamma \max_a P(a)\right)$$
$$P(\mu^{n+1} > \mu^n) \sim U\big(0, P(a)\big) \tag{9}$$

Considering the policy in (8), the assignment of rewards is asymmetric. In particular, for a single time window n, trials in which the force allows for an increment in performance weigh more than trials in which the performance worsens. Since positive rewards have the effect of incrementing the probability to reduce the step size in time, the policy explicitly favors convergence over exploration through time. After updating its status, the algorithm draws the action a with uniform probability in the interval 0 and *P(a)* and updates the step size accordingly:

$$\mu^{n+1} = \mu^n + \Delta\mu^n$$
$$\Delta\mu^n := \begin{cases} 1.1 \cdot \mu^n, & if\ a = 'increment' \\ 0.9 \cdot \mu^n, & if\ a = 'decrement' \end{cases} \tag{10}$$

The values of the parameters α and γ were determined by simulating the behavior of the model in the presence of a specific constant reward, given the initial conditions of $P(a)^0 = 0$, and $\mu^0 = 1N$. In particular, given a constant reward of 1, the algorithm is required to reduce the step up to 95% on average after 2 movement sets (10 steps of update along each direction). Consistently, when considering a constant reward of -0.5, the algorithm should be able to increase the step value up to 50% within a movement set. Finally, given a reward of 0, the value of the step should be constant except for a random contribution of Q ≤ 30%, to allow for small fluctuations around the threshold. The conditions above can be satisfied by setting α = 0.3 and γ = 0.5.

References

Bohannon, R. W., and Smith, M. B. (1987). Interrater reliability of a modified Ashworth scale of muscle spasticity. *Phys. Ther.* 67, 206–7. Available at: http://www.ncbi.nlm.nih.gov/pubmed/3809245 [Accessed April 13, 2014].

Bowerman, C. C., Semrau, J. A., Kiss, Z., and Dukelow, S. P. (2012). The Importance of Somatosensory Deficits in Neurological Disease. in *International Functional Elsctrical Stimulation Society (IFESS) Conference* (Banff, Alberta, Canada,September 9-12, 2012), 2–5.

Carey, L. M., Matyas, T. A., and Oke, L. E. (1993). Sensory loss in stroke patients: effective training of tactile and proprioceptive discrimination. *Arch. Phys. Med. Rehabil.* 74, 602–11. Available at: http://www.ncbi.nlm.nih.gov/pubmed/8503750 [Accessed April 17, 2014].

Casadio, M., Sanguineti, V., Morasso, P. G., and Arrichiello, V. (2006). Braccio di Ferro: a new haptic workstation for neuromotor rehabilitation. *Technol. Health Care* 14, 123–42. Available at: http://www.ncbi.nlm.nih.gov/pubmed/16971753 [Accessed February 13, 2014].

Chieffo, R., Inuggi, A., Straffi, L., Coppi, E., Gonzalez-Rosa, J., Spagnolo, F., Poggi, A., Comi, G., Comola, M., and Leocani, L. (2013). Mapping early changes of cortical motor output after subcortical stroke: a transcranial magnetic stimulation study. *Brain Stimul.* 6, 322–9. doi:10.1016/j.brs.2012.06.003.

Connell, L. A., Lincoln, N. B., and Radford, K. A. (2008). Somatosensory impairment after stroke: frequency of different deficits and their recovery. *Clin. Rehabil.* 22, 758–67. doi:10.1177/0269215508090674.

Corbetta, M., and Shulman, G. L. (2011). Spatial neglect and attention networks. *Annu. Rev. Neurosci.* 34, 569–99. doi:10.1146/annurev-neuro-061010-113731.

Darainy, M., Vahdat, S., and Ostry, D. J. (2013). Perceptual learning in sensorimotor adaptation. *J. Neurophysiol.* 110, 2152–62. doi:10.1152/jn.00439.2013.

Dipietro, L., Krebs, H. I., Volpe, B. T., Stein, J., Bever, C., Mernoff, S. T., Fasoli, S. E., and Hogan, N. (2012). Learning, not adaptation, characterizes stroke motor recovery: evidence from kinematic changes induced by robot-assisted therapy in trained and untrained task in the same workspace. *IEEE Trans. Neural Syst. Rehabil. Eng.* 20, 48–57. doi:10.1109/TNSRE.2011.2175008.

Doyle, S., Bennett, S., Fasoli, S. E., and McKenna, K. T. (2010). Interventions for sensory impairment in the upper limb after stroke. *Cochrane database Syst. Rev.*, CD006331. doi:10.1002/14651858.CD006331.pub2.

Dukelow, S. P., Herter, T. M., Bagg, S. D., and Scott, S. H. (2012). The independence of deficits in position sense and visually guided reaching following stroke. *J. Neuroeng. Rehabil.* 9, 72. doi:10.1186/1743-0003-9-72.

Dukelow, S. P., Herter, T. M., Moore, K. D., Demers, M. J., Glasgow, J. I., Bagg, S. D., Norman, K. E., and Scott, S. H. (2010). Quantitative assessment of limb position sense following stroke. *Neurorehabil. Neural Repair* 24, 178–87. doi:10.1177/1545968309345267.

Elangovan, N., Herrmann, A., and Konczak, J. (2014). Assessing Proprioceptive Function: Evaluating Joint Position Matching Methods Against Psychophysical Thresholds. *Phys. Ther.* doi:10.2522/ptj.20130103.

Fisher, B. E., and Sullivan, K. J. (2001). Activity-dependent factors affecting poststroke functional outcomes. *Top. Stroke Rehabil.* 8, 31–44. Available at: http://www.ncbi.nlm.nih.gov/pubmed/14523736 [Accessed November 22, 2014].

Frassinetti, F., Rossi, M., and Làdavas, E. (2001). Passive limb movements improve visual neglect. *Neuropsychologia* 39, 725–33. Available at: http://www.ncbi.nlm.nih.gov/pubmed/11311302.

Fugl-Meyer, A. R., Jääskö, L., Leyman, I., Olsson, S., and Steglind, S. (1975). The post-stroke hemiplegic patient. 1. a method for evaluation of physical performance. *Scand. J. Rehabil. Med.* 7, 13–31. Available at: http://www.ncbi.nlm.nih.gov/pubmed/1135616 [Accessed April 15, 2014].

Gandevia, S. C., McCloskey, D. I., and Burke, D. (1992). Kinaesthetic signals and muscle contraction. *Trends Neurosci.* 15, 62–5. Available at: http://www.ncbi.nlm.nih.gov/pubmed/1374964 [Accessed September 11, 2014].

Ghez, C., Gordon, J., Ghilardi, M. F., and Sainburg, R. L. (1995). "Contributions of vision and proprioception to accuracy in limb movements.," in *The cognitive neurosciences,* ed. M. S. Gazzaniga (Cambridge, MA, US: The MIT Press), 549–564.

Goble, D. J., and Brown, S. H. (2007). Task-dependent asymmetries in the utilization of proprioceptive feedback for goal-directed movement. *Exp. Brain Res.* 180, 693–704. doi:10.1007/s00221-007-0890-7.

Gordon, J., Ghilardi, M. F., and Ghez, C. (1995). Impairments of reaching movements in patients without proprioception. I. Spatial errors. *J. Neurophysiol.* 73, 347–60. Available at: http://www.ncbi.nlm.nih.gov/pubmed/7714577 [Accessed February 3, 2014].

Han, C. E., Arbib, M. A., and Schweighofer, N. (2008). Stroke rehabilitation reaches a threshold. *PLoS Comput. Biol.* 4, e1000133. doi:10.1371/journal.pcbi.1000133.

Iandolo, R., Squeri, V., De Santis, D., Morasso, P., and Casadio, M. (2014). Testing proprioception in intrinsic and extrinsic coordinate systems: is there a difference? in *5th IEEE RAS & EMBS International Conference on Biomedical Robotics and Biomechatronics (BioRob)* (August 12-15, 2014, São Paulo, Brazil), 6961–6964.

Kantak, S. S., Jones-Lush, L. M., Narayanan, P., Judkins, T. N., and Wittenberg, G. F. (2013). Rapid plasticity of motor corticospinal system with robotic reach training. *Neuroscience* 247, 55–64. doi:10.1016/j.neuroscience.2013.05.001.

Kerkhoff, G., and Rossetti, Y. (2006). Plasticity in spatial neglect: recovery and rehabilitation. *Restor. Neurol. Neurosci.* 24, 201–6. Available at: http://www.ncbi.nlm.nih.gov/pubmed/17119298 [Accessed September 8, 2014].

Kusoffsky, A., Wadell, I., and Nilsson, B. Y. (1982). The relationship between sensory impairment and motor recovery in patients with hemiplegia. *Scand. J. Rehabil. Med.* 14, 27–32. Available at: http://www.ncbi.nlm.nih.gov/pubmed/7063817 [Accessed August 14, 2014].

Leibowitz, N., Levy, N., Weingarten, S., Grinberg, Y., Karniel, A., Sacher, Y., Serfaty, C., and Soroker, N. (2008). Automated measurement of proprioception following stroke. *Disabil. Rehabil.* 30, 1829–36. doi:10.1080/09638280701640145.

Lincoln, N., Jackson, J., and Adams, S. (1998). Reliability and Revision of the Nottingham Sensory Assessment for Stroke Patients. *Physiotherapy* 84, 358–365. doi:10.1016/S0031-9406(05)61454-X.

Meyer, S., Karttunen, A. H., Thijs, V., Feys, H., and Verheyden, G. (2014). How do somatosensory deficits in the arm and hand relate to upper limb impairment, activity, and participation problems after stroke? A systematic review. *Phys. Ther.* 94, 1220–31. doi:10.2522/ptj.20130271.

Milot, M.-H., Spencer, S. J., Chan, V., Allington, J. P., Klein, J., Chou, C., Pearson-Fuhrhop, K., Bobrow, J. E., Reinkensmeyer, D. J., and Cramer, S. C. (2014). Corticospinal excitability as a predictor of functional gains at the affected upper limb following robotic training in chronic stroke survivors. *Neurorehabil. Neural Repair* 28, 819–27. doi:10.1177/1545968314527351.

Morasso, P. (2013). "Towards an Integrated Approach to Multimodal Assistance of Stroke Patients Based on the Promotion of Intentionality," in *Converging Clinical and Engineering Research on*

Neurorehabilitation Biosystems & Biorobotics., eds. J. L. Pons, D. Torricelli, and M. Pajaro (Berlin, Heidelberg: Springer Berlin Heidelberg), 23–27. doi:10.1007/978-3-642-34546-3.

Nasir, S. M., Darainy, M., and Ostry, D. J. (2013). Sensorimotor adaptation changes the neural coding of somatosensory stimuli. *J. Neurophysiol.* 109, 2077–85. doi:10.1152/jn.00719.2012.

Ostry, D. J., Darainy, M., Mattar, A. A. G., Wong, J., and Gribble, P. L. (2010). Somatosensory plasticity and motor learning. *J. Neurosci.* 30, 5384–93. doi:10.1523/JNEUROSCI.4571-09.2010.

Pellegrino, G., Tomasevic, L., Tombini, M., Assenza, G., Bravi, M., Sterzi, S., Giacobbe, V., Zollo, L., Guglielmelli, E., Cavallo, G., et al. (2012). Inter-hemispheric coupling changes associate with motor improvements after robotic stroke rehabilitation. *Restor. Neurol. Neurosci.* 30, 497–510. doi:10.3233/RNN-2012-120227.

Proske, U., and Gandevia, S. C. (2009). The kinaesthetic senses. *J. Physiol.* 587, 4139–46. doi:10.1113/jphysiol.2009.175372.

Rand, D., Weiss, P. L. (Tamar), and Gottlieb, D. (1999). Does Proprioceptive Loss Influence Recovery of the Upper Extremity After Stroke? *Neurorehabil. Neural Repair* 13, 15–21. doi:10.1177/154596839901300104.

Reinhart, S., Schmidt, L., Kuhn, C., Rosenthal, A., Schenk, T., Keller, I., and Kerkhoff, G. (2012). Limb activation ameliorates body-related deficits in spatial neglect. *Front. Hum. Neurosci.* 6, 188. doi:10.3389/fnhum.2012.00188.

Robertson, I. H., and North, N. (1993). Active and passive activation of left limbs: influence on visual and sensory neglect. *Neuropsychologia* 31, 293–300. Available at: http://www.ncbi.nlm.nih.gov/pubmed/8492882 [Accessed September 21, 2014].

De Santis, D., Zenzeri, J., Casadio, M., Masia, L., Morasso, P. G., and Squeri, V. (2014a). A new method for evaluating kinesthetic acuity during haptic interaction. *Robotica* published , 1–16. doi:doi:10.1017/S0263574714002252.

De Santis, D., Zenzeri, J., Masia, L., Squeri, V., and Morasso, P. (2014b). Exploiting the Link between Action and Perception : Minimally Assisted Robotic Training of the Kinesthetic Sense. in *5th IEEE RAS & EMBS International Conference on Biomedical Robotics and Biomechatronics (BioRob)* (August 12-15, 2014, São Paulo, Brazil), 287–292.

Schabrun, S. M., and Hillier, S. (2009). Evidence for the retraining of sensation after stroke: a systematic review. *Clin. Rehabil.* 23, 27–39. doi:10.1177/0269215508098897.

Scheidt, R. A., Lillis, K. P., and Emerson, S. J. (2010). Visual, motor and attentional influences on proprioceptive contributions to perception of hand path rectilinearity during reaching. *Exp. brain Res.* 204, 239–54. doi:10.1007/s00221-010-2308-1.

Semrau, J. A., Herter, T. M., Scott, S. H., and Dukelow, S. P. (2013). Robotic identification of kinesthetic deficits after stroke. *Stroke* 44, 3414–21. doi:10.1161/STROKEAHA.113.002058.

Simo, L. S., Botzer, L., Ghez, C., and Scheidt, R. A. (2014). A robotic test of proprioception within the hemiparetic arm post-stroke. *J. Neuroeng. Rehabil.* 11, 77. doi:10.1186/1743-0003-11-77.

Smith, D. L., Akhtar, A. J., and Garraway, W. M. (1983). Proprioception and spatial neglect after stroke. *Age Ageing* 12, 63–9. Available at: http://www.ncbi.nlm.nih.gov/pubmed/6846094 [Accessed September 9, 2014].

Squeri, V., Zenzeri, J., Morasso, P., and Basteris, A. (2011). Integrating proprioceptive assessment with proprioceptive training of stroke patients. *IEEE Int. Conf. Rehabil. Robot.* 2011, 5975500. doi:10.1109/ICORR.2011.5975500.

Sullivan, J. E., and Hedman, L. D. (2008). Sensory dysfunction following stroke: incidence, significance, examination, and intervention. *Top. Stroke Rehabil.* 15, 200–17. doi:10.1310/tsr1503-200.

Taub, E., and Berman, A. J. (1963). Avoidance conditioning in the absence of relevant proprioceptive and exteroceptive feedback. *J. Comp. Physiol. Psychol.* 56, 1012–6. Available at: http://www.ncbi.nlm.nih.gov/pubmed/14100937 [Accessed September 9, 2014].

Turner, D. L., Ramos-Murguialday, A., Birbaumer, N., Hoffmann, U., and Luft, A. (2013). Neurophysiology of robot-mediated training and therapy: a perspective for future use in clinical populations. *Front. Neurol.* 4, 184. doi:10.3389/fneur.2013.00184.

Tyson, S. F., Hanley, M., Chillala, J., Selley, A. B., and Tallis, R. C. (2008). Sensory loss in hospital-admitted people with stroke: characteristics, associated factors, and relationship with function. *Neurorehabil. Neural Repair* 22, 166–72. doi:10.1177/1545968307305523.

Vahdat, S., Darainy, M., and Ostry, D. J. (2014). Structure of plasticity in human sensory and motor networks due to perceptual learning. *J. Neurosci.* 34, 2451–63. doi:10.1523/JNEUROSCI.4291-13.2014.

De Vignemont, F. (2010). Body schema and body image - Pros and cons. *Neuropsychologia* 48, 669–680. doi:10.1016/j.neuropsychologia.2009.09.022.

Van Vleet, T. M., Hoang-duc, A. K., DeGutis, J., and Robertson, L. C. (2011). Modulation of non-spatial attention and the global/local processing bias. *Neuropsychologia* 49, 352–9. doi:10.1016/j.neuropsychologia.2010.11.021.

Wong, J. D., Kistemaker, D. A., Chin, A., and Gribble, P. L. (2012). Can proprioceptive training improve motor learning? *J. Neurophysiol.* 108, 3313–21. doi:10.1152/jn.00122.2012.

Yarossi, M., Adamovich, S., and Tunik, E. (2014). Sensorimotor Cortex Reorganization in Subacute and Chronic Stroke: A Neuronavigated TMS Study. in *36th Annual International Conference of the IEEE Engineering in Medicine and Biology Society*, 5788–5791. Available at: http://emb.citengine.com/event/embc-2014/paper-details?pdID=2080 [Accessed September 8, 2014].
